# Research into performance optimization control strategy of a hydrostatic drive bulldozer based on the constant speed cruise

**DOI:** 10.1038/s41598-025-08019-w

**Published:** 2025-07-27

**Authors:** Hongbin Qiang, He Li, Shaopeng Kang, Kailei Liu, Jing Yang, Xingfei Luo

**Affiliations:** 1 School of Mechanical Engineering, Jiangsu University of Technology, Changzhou, 213001 China; 2 Jiangsu Provicial Engineering Research Center for Advanced Fluid Power and Equipment, Changzhou, 213001 China; 3 Liugong Changzhou Machinery co., LTD, Changzhou, 213001 China

**Keywords:** Bulldozer, Hydrostatic transmission, Constant speed cruise, Performance optimization, Throttle and displacement coordinated control, Fuel efficiency, Co-simulation, Engineering, Mechanical engineering

## Abstract

Hydrostatic bulldozers, mainly medium- and low-horsepower machines, are widely used in engineering, and improving their fuel economy is crucial under the global push for energy conservation. To address the poor fuel economy of the bulldozer during repetitive, precision tasks or idle transfers, a performance optimization control strategy (POCS) based on constant speed cruise(CSC) is designed. This strategy achieves optimal overall machine efficiency under cruise control by adjusting the engine throttle and the displacement of the hydraulic pump and motor. On one hand, engine performance is optimized by designing the operating modes and the optimal fuel consumption line based on the engine’s general characteristic curve. The engine operating point is switched according to this line, reducing fuel consumption. On the other hand, the hydraulic system performance is optimized by proposing a method for determining the optimal efficiency combination for the pump and motor, considering the efficiencies of the engine, pump, and motor. This method calculates the required engine power and the preset motor speed to determine the displacement settings of the pump and motor at maximum efficiency for each operating condition. The required engine power is derived by reversing the efficiency, while maintaining the motor speed (i.e., working point identification).Then, the engine and hydraulic system models are developed and co-simulated. The results show that, compared to the constant-speed control strategy at 1600 rpm and 1700 rpm, the POCS results in a more concentrated distribution of working points, with overall fuel consumption reduced by 6.5% and 3.7%, respectively. In the final experiment, compared with the 1700 rpm constant speed control strategy (CSCS), the total fuel consumption was reduced by 4.1%. This control strategy offers a novel and effective approach to optimizing bulldozer fuel consumption and provides valuable insights for other hydrostatic applications.

## Introduction

As a common type of construction machinery, bulldozers are widely used in construction, agriculture, municipal projects, and mining for tasks such as excavation and leveling^1^. Against the backdrop of global resource scarcity and the push for intelligent and unmanned operations, construction machinery must not only ensure stable operation but also achieve advanced energy-saving technology, which has become a critical measure of core competitiveness^2,3^. Therefore, improving the fuel economy of bulldozers and achieving precise operational speeds are of paramount importance^4,5^.

Focusing on energy efficiency, performance optimization of construction machinery in recent years has primarily targeted power matching between the engine and variable loads. In the field of hydrostatic transmission, the most common approach is controlling the engine to maintain fixed operating points. He proposed a control strategy combining constant power and variable power. By adjusting the pump displacement, this strategy rapidly stabilizes engine speed by regulating the torque absorbed by the pump^6^. Gao established engine operating points in stages according to the work cycle, combining torque-sensing control and speed-sensing control to ensure stable operating points during the same work phase^7^. Choi and Kim introduced hybrid power systems, incorporating an electric motor into the drivetrain. By controlling the motor’s torque, they achieved peak shaving and valley filling for load torque, thereby maintaining the engine’s operating points within the economic operating zone^8,9^.

To ensure the stability and precision of bulldozer operations, cruise control functionality is the optimal solution and forms the foundation for unmanned mechanical operations^10,11^. For hydrostatic vehicles, the integration of throttle and hydraulic system control based on load conditions represents the current research trend globally. Wang designed a throttle pedal adjustment mechanism for the Lovol TA800 tractor and developed a longitudinal dynamic model. By adjusting the throttle opening under preset gear positions, they achieved cruise control^12^. He coupled the controls of the throttle pedal and speed pedal into a single variable, simplifying speed regulation. Using an expert PID control algorithm and GNSS speed signal feedback, they realized cruise control for rice transplanters^13^. Zhao designed throttle and load adjustment mechanisms for hydrostatic tractors, utilizing coordinated throttle and displacement control to regulate vehicle speed^5^. Coen developed a cruise control system for the New Holland hydrostatic combine harvester, where the engine provided power to a variable hydraulic pump, which then transmitted power to a hydraulic motor. The hydraulic flow rate was determined by the engine speed and the swash plate angle of the variable pump. Through different combinations of engine speed and pump swash plate angle, they achieved consistent operational speeds while reducing engine RPM^14^.

Based on the above research, two aspects warrant further investigation:

1. In the CSC mode, it is crucial for the hydraulic system to absorb engine output power appropriately. Insufficient power absorption may result in the inability to maintain the desired vehicle speed, while excessive power absorption can lead to energy waste. Therefore, performance optimization for engineering vehicles should focus on maximizing overall system efficiency by ensuring both engine fuel economy and hydraulic system efficiency. Hydraulic system performance optimization is particularly critical and requires a comprehensive consideration of the efficiency of the engine, hydraulic pump, and hydraulic motor. Ultimately, the effectiveness of fuel economy should be reflected on the engine’s universal characteristic curve to validate the control strategy’s feasibility.
2. For power matching, while fixed operating point control strategies can avoid high fuel consumption zones in most cases, they may cause the operating point to shift into less efficient regions when external load variations require different torques. On the other hand, the staged operating point switching control method is inherently a real-time switching strategy at the macro level. In specific CSC conditions, a Performance Optimization Strategy that adjusts the operating point along the optimal fuel consumption curve based on variable loads in real-time can be effectively applied to engine performance optimization.

This study aims to design a Performance Optimization Strategy for CSC conditions, adopting a throttle-displacement coordinated control approach. It optimizes the efficiency of both the engine and the hydraulic system, ensuring overall performance optimization for hydrostatic bulldozers while maintaining operational efficiency. This strategy aligns with modern industrial demands for high-efficiency and precision operations and provides valuable insights for fuel economy optimization in hydrostatic-driven vehicles and similar engineering machinery.

## System operating characteristics analysis

### CSC function characteristics analysis

Cruise control is widely used in automobiles to reduce driver fatigue during long highway drives. Similarly, in bulldozer operations like trenching, soil pushing, or traveling unloaded to a new site, the tasks involve prolonged flat-ground operations, large-scale earthmoving, and long-distance hauling. These conditions demand steady speeds and high efficiency, with gradual rather than abrupt load fluctuations.

To ensure a high degree of consistency and precision in construction processes, relying solely on the driver’s extensive experience is often insufficient. With the cruise control function, operators no longer need to control the engine state manually by continuously pressing the throttle pedal. When external forces affect the vehicle’s motion, the system can automatically adjust acceleration through the cruise control mechanism^15^. Thus, in the aforementioned specific application scenarios, the use of cruise control enables bulldozers to perform tasks more efficiently and smoothly, ensuring consistent and stable operation.

### Analysis of engine operating characteristics

The diesel engine, as the power source for bulldozers, plays a crucial role in evaluating whether it meets the vehicle’s power and fuel economy require-ments^16^. In the collaborative project, the bulldozer is equipped with the Cummins QSL9.3 electronically controlled engine, with its technical parameters shown in Table 1.

**Table 1:** The bulldozer and its engine specifications.

Bulldozer weight (kg)	20,000
Bulldozer track radius (m)	0.45
Bulldozer transmission ratio	54.7
Engine type	Vertical, inline, water-cooled, six-cylinder
Engine air intake	Turbocharged, air-to-air intercooler
Engine displacement (L)	9.3
Engine bore (mm)	116.5
Engine rated power (kw)	162 ~ 180
Engine maximum torque (N·m)	1187
Engine fuel system	High-pressure common rail

**Fig. 1:**
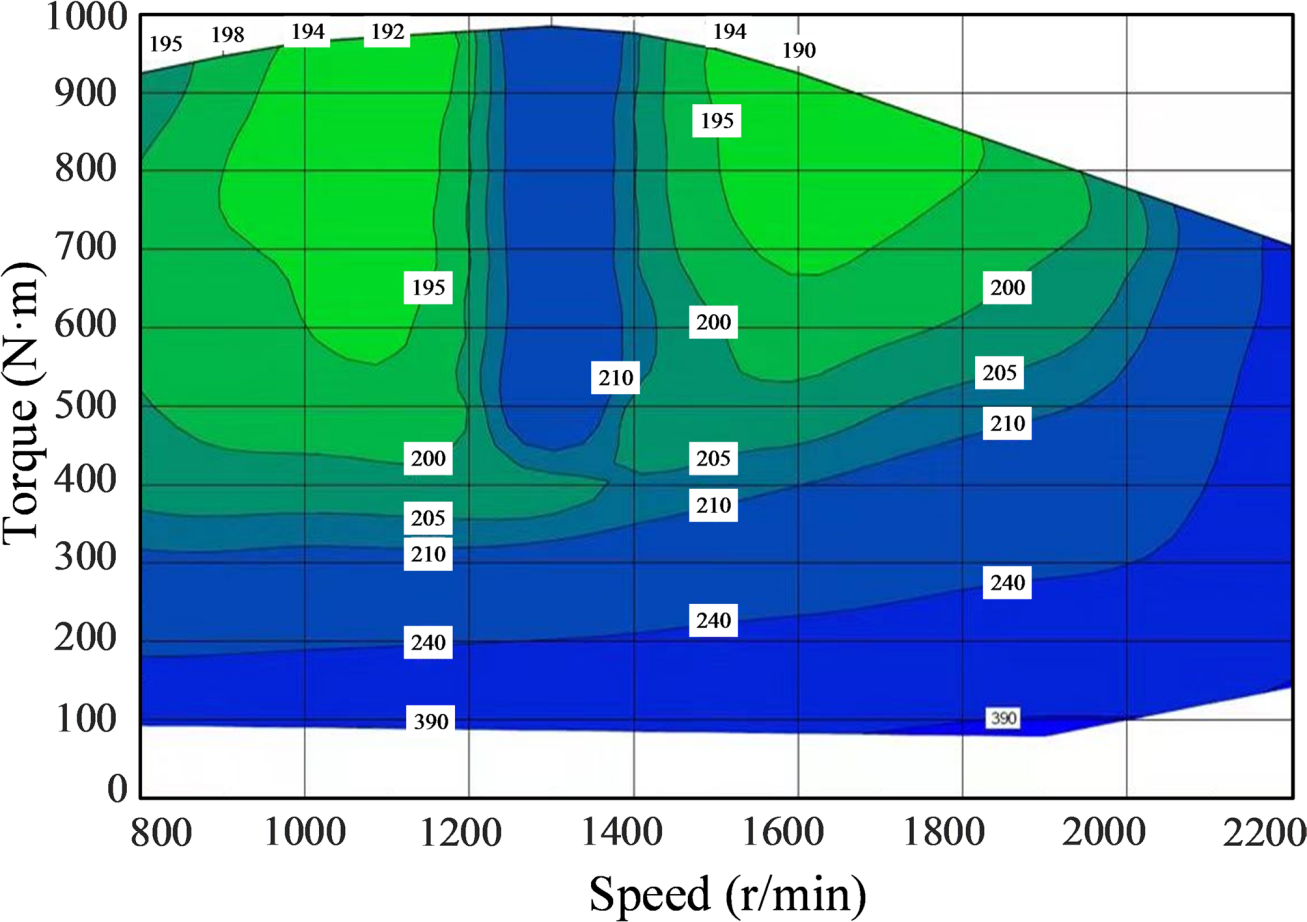
Universal characteristic curve of an engine.

The universal characteristic curve comprehensively displays the dynamic performance parameters of the engine, including key features such as speed, torque, and fuel consumption rate. By analyzing the universal characteristic curve, one can determine the engine’s optimal operating range, thereby improving fuel efficiency and performance, while reducing emissions and wear^17^. The universal characteristic curve of this engine is shown in Fig. 1. Under the same power, there are multiple working points with different speed and torque combinations. Each working point corresponds to a different fuel consumption rate, where a lower fuel consumption rate indicates better fuel efficiency of the engine^18^. The two light green areas in the graph represent the optimal economic operating range, indicating that the engine operates within the lowest fuel consumption zone both at low power and near the rated power.

### Analysis of hydraulic transmission system operating characteristics

The hydrostatic transmission has a simple structure, can achieve stepless speed variation, has high transmission efficiency, and is easy to control, making it widely used in bulldozers^19,20^. The hydraulic transmission system is the core of the hydrostatic bulldozer, and its performance directly affects the overall performance and work efficiency of the bulldozer^21^.

**Fig. 2:**
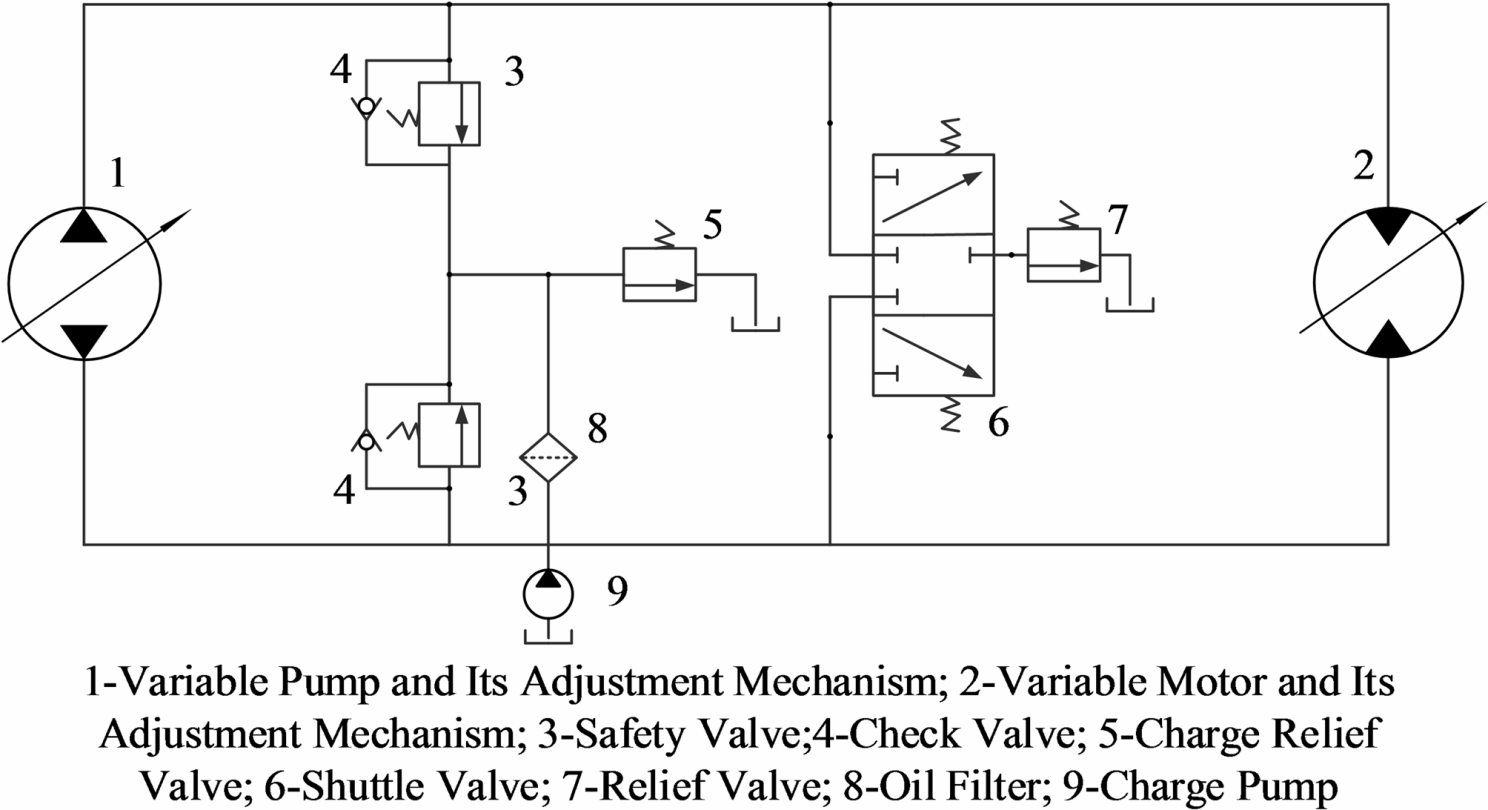
Hydraulic drive system unilateral circuit.

As shown in Fig. 2, the single-side circuit of the hydraulic transmission system for the bulldozer primarily consists of fundamental components such as a hydraulic pump, hydraulic motor, safety valves, charge pump, and shuttle valve. Being a closed-loop system, the high-pressure and low-pressure oil circuits interchange during operation. Therefore, two safety valves are installed to ensure the system’s maximum pressure is maintained at 45 MPa.

The pumps and motors used in the cooperative project are both bidirectional variable units. These not only adapt to complex and variable operational demands but also improve system efficiency. Their specific parameters are listed in Table 2.

**Table 2:** Pumps and motors technical data.

Parameter name	Hydraulic component	
	Pump	Motor
Type	A24VG125	A6VE215
Displacement (L)	125	216.5
Rated speed (r/min)	3000	2999

## CSC control

### Principle of CSC control

In the hydraulic transmission system section, the flow rate generated by the hydraulic pump is:

1 $${q_p} = {V_p}{n_p}{\eta _p}_m$$

where *η*_*pm*_ represents the speed of the pump; *q*_*P*_ represents the flow rate generated by the pump, *η*_*p*_ represents the volumetric efficiency of the pump, and *V*_*P*_ represents the displacement of the pump.

For the hydraulic motor, the calculation formula for its speed is:

2 $${n_v} = \frac{{{q_t}}}{{{V_v}}} = \frac{{{q_v}{\eta _{vm}}}}{{{V_v}}}$$

where *η*_*v*_ represents the motor speed; *q*_*t*_ represents the theoretical flow received by the motor; *q*_*v*_ represents the actual flow received by the motor; *η*_*vm*_ represents the total efficiency of the motor; and *V*_*v*_ represents the displacement of the motor.

Due to losses such as friction and heat generation in the pipes, the flow rate in the system can be expressed as:

3 $${q_v} = {q_p}{\eta _s}$$

where *η*_*s*_ represents the total efficiency of the system.

By solving Eqs. (9) to (11) simultaneously, the motor speed can be expressed as:

4 $${n_v} = \frac{{{V_p}{n_p}{\eta _{pm}}{\eta _{vm}}{\eta _s}}}{{{V_v}}}$$

**Fig. 3:**
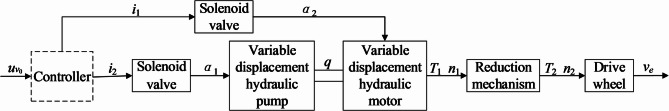
Target speed control strategy.

It is evident that, under varying engine speeds, to achieve constant speed cruising, the motor speed (and thus the vehicle speed) can be stabilized by adjusting the displacement of the hydraulic pump and motor. Figure 3 shows the control block diagram for implementing constant speed cruising in the bulldozer. After setting an ideal vehicle speed, the controller sends current to the electronically controlled proportional valve, which then adjusts the displacement of the pump and motor, regulating the flow within the system.

### Throttle-displacement coordinated control

**Fig. 4:**
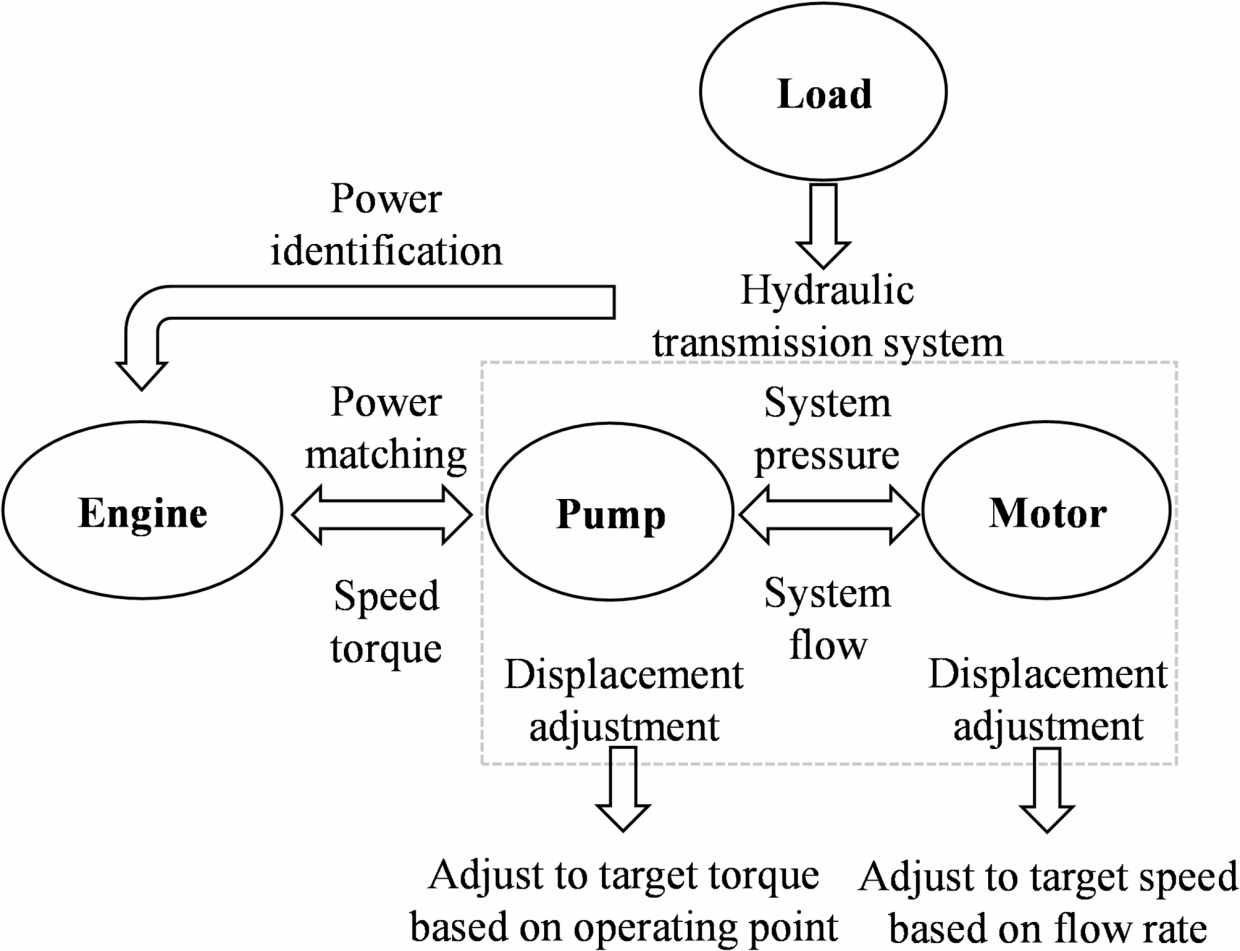
Working principle and interrelationship of the main components of a bulldozer.

The hydrostatic bulldozer system includes components like the engine, hydraulic pump, and hydraulic motor, which work together to match and adjust power during variable load operations. The engine adjusts its output power and speed based on load demands, transmitting power to the hydraulic system. The hydraulic pump regulates pressure and flow to meet changing load requirements, while the hydraulic motor adjusts its speed based on the pump’s flow. By controlling the displacement of the pump and motor, the engine’s output power aligns with the load, preventing energy waste and overload. As shown in Fig. 4, the working principle and relationships of the main components are depicted. To ensure efficient operation and precise control of the entire system, this study proposes a overall control strategy for the bulldozer: the throttle-displacement coordinated control strategy. This strategy further optimizes system performance by dynamically adjusting the hydraulic pump displacement and engine throttle opening according to engine speed and load requirements.

**Fig. 5:**
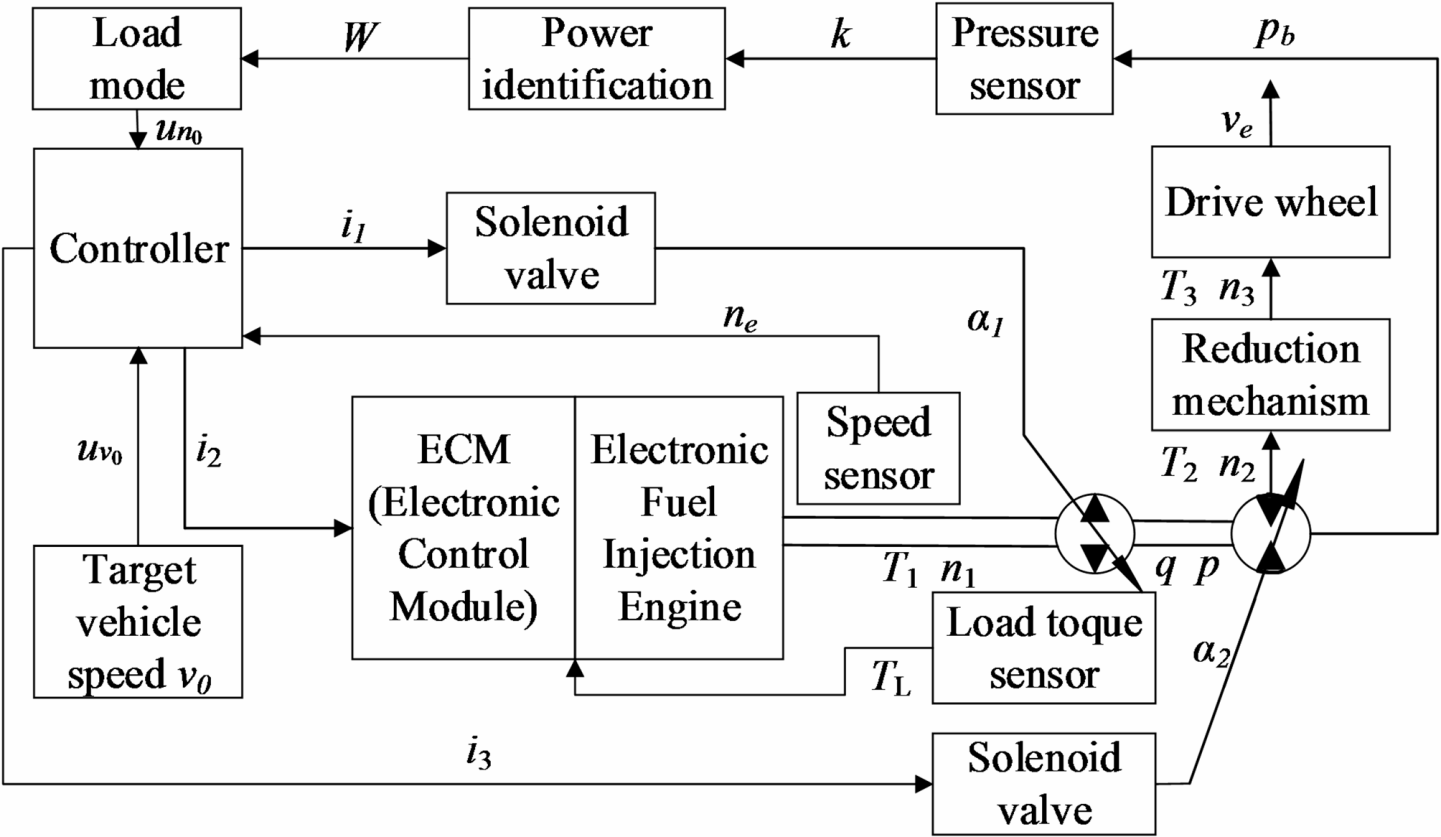
Bulldozer overall control strategy.

Based on the preceding content, the overall control strategy block diagram is established as shown in Fig. 5. The controller in the diagram consists of three main control modules: engine throttle control, hydraulic pump displacement control, and hydraulic motor displacement control, enabling coordinated control of throttle and displacement.

## POCS based on CSC

### Engine performance optimization

#### Plotting the optimal fuel consumption line

Based on the universal characteristics of the engine, it is known that the working point where the constant power line and the constant fuel consumption rate line are tangent represents the optimal energy-saving point. When the engine operates at the optimal energy-saving point, the fuel consumption is minimized for completing the same amount of work. By connecting all the optimal energy-saving points with a curve, we obtain the optimal fuel consumption line of the diesel engine.

**Fig. 6:**
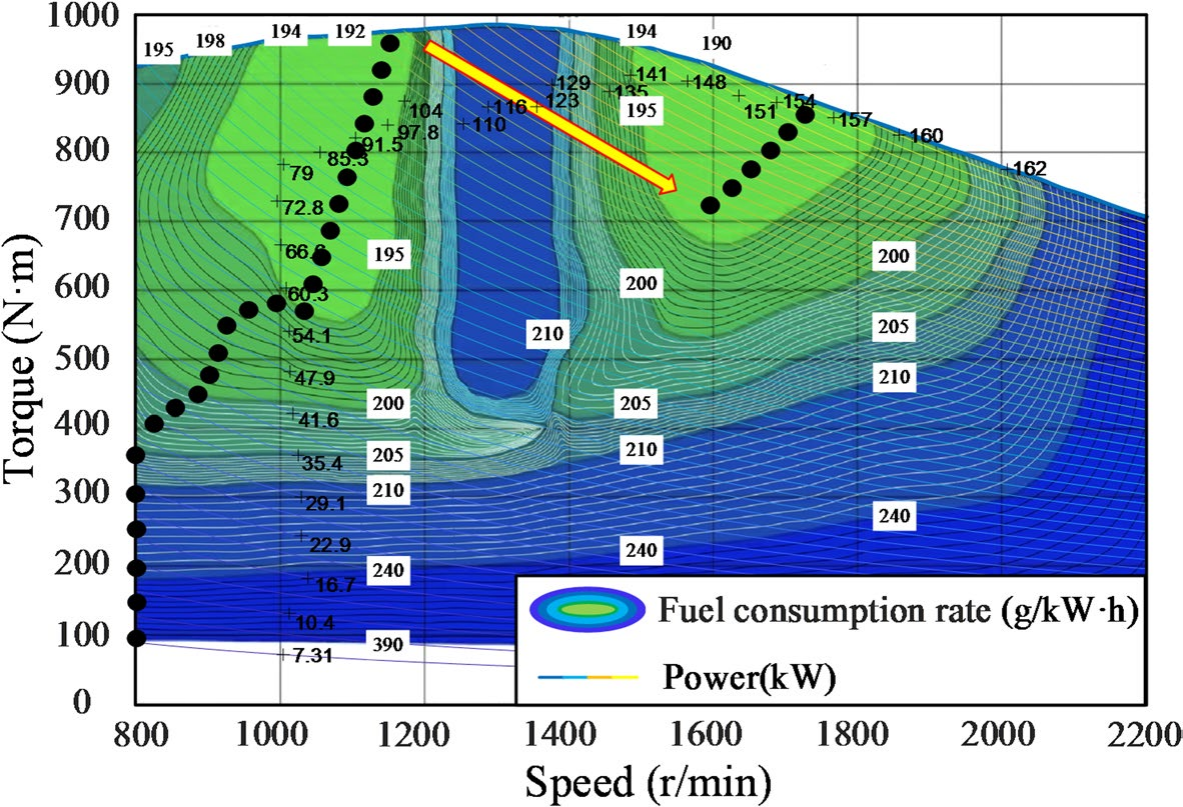
Schematic of optimal fuel consumption line finding.

Since Fig. 1 only includes speed, torque, and fuel consumption rate, to facilitate finding the tangent points, it is necessary to incorporate the engine’s constant power curves. The three types of special points are extracted and organized into three columns of data. Then, the extracted data are fitted, and the fitted constant power curves are subdivided. After that, the constant fuel consumption curves are also subdivided. Finally, the program reads the original diagram and overlays the two images. The final result is shown in Fig. 6.

**Fig. 7:**
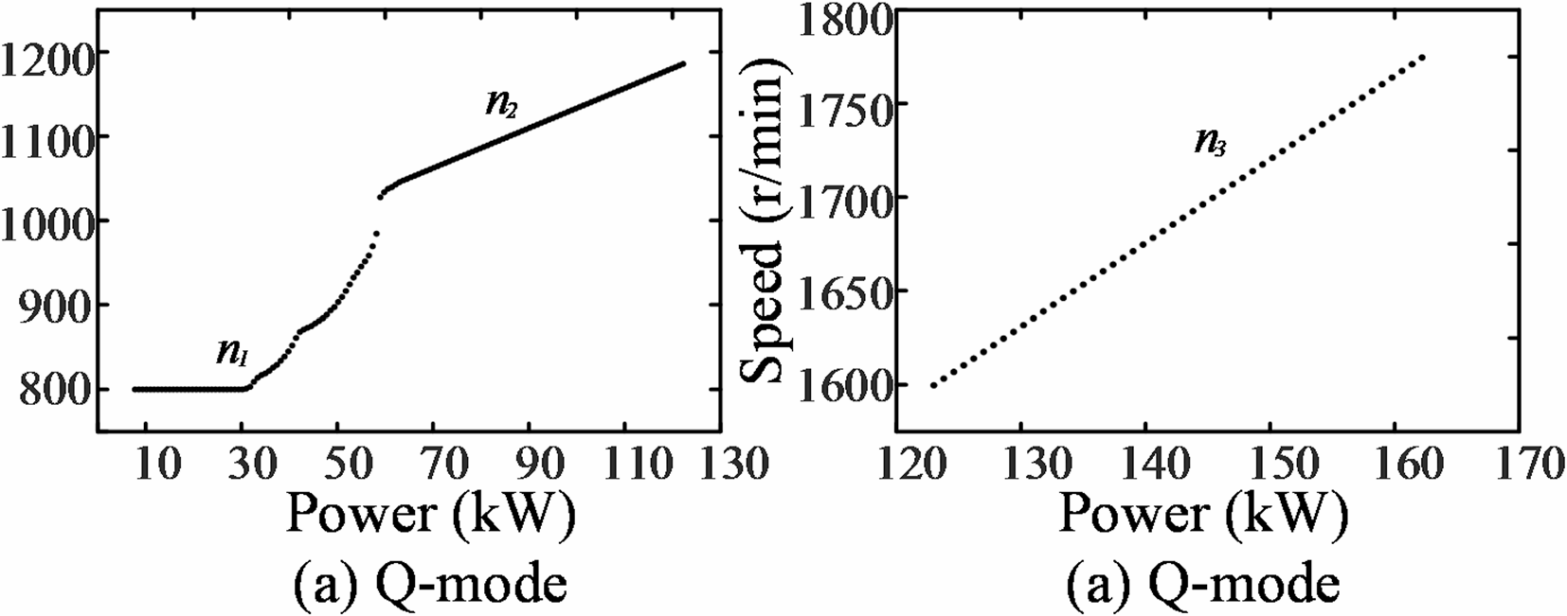
Q and Z mode power-speed curve.

Observing the constant power curves in Fig. 6 reveals that when the engine power rises to approximately 123 kW, the operating point on the left side is no longer within the region of minimum fuel consumption. However, by following the constant power curve toward the lower-right corner, it can be observed that this power level intersects with the region of minimum fuel consumption on the right side. Additionally, power levels greater than 123 kW but less than the rated power also pass through this region. To maintain operating points within this power range while simplifying the control function, power and speed can be approximated by a fixed linear relationship. After identifying the tangents to the constant power and fuel consumption curves in Fig. 6, the corresponding speed values for each power segment are collected. These data points are then plotted in a scatter plot with power on the horizontal axis and speed on the vertical axis, as shown in Fig. 7, to analyze their characteristics.

During the transition of the operating point under the aforementioned 123 kW condition, the engine speed needs to switch from approximately 1185 r/min to 1600 r/min. Regardless of whether the power increases or decreases, the speed undergoes a sharp change, posing challenges such as increased mechanical stress, reduced combustion efficiency, and severe vibration.

Based on the above analysis, the power modes are divided into two categories: light-load mode (Q mode) and heavy-load mode (Z mode), corresponding to the left and right sections of the curve. In Fig. 7a, there are two notable inflection points that divide the curve into three segments. To ensure greater precision when invoking the optimal fuel consumption line, a holistic fitting approach is avoided. Instead, only the nonlinear segment in Fig. 7a is fitted to generate a 2-D lookup table, which is integrated into the engine model for simulation purposes.The first and third segments of Fig. 7a, similar to the curve in Fig. 7b, can directly represent the relationship between power and speed using linear function expressions, defined as follows:

5 $${n_1} = 800$$

6 $${n_2} = 2.39{P_2} + 893.72$$

7 $${n_3} = 4.46{P_3} + 1051.12$$

where *n*_*1*_,*n*_*2*_ *and n*_*3*_ represent the speed of the engine; *P*_*2*_ and *P*_*3*_ represent the power of the engine.

#### Operating point switching control

**Fig. 8:**
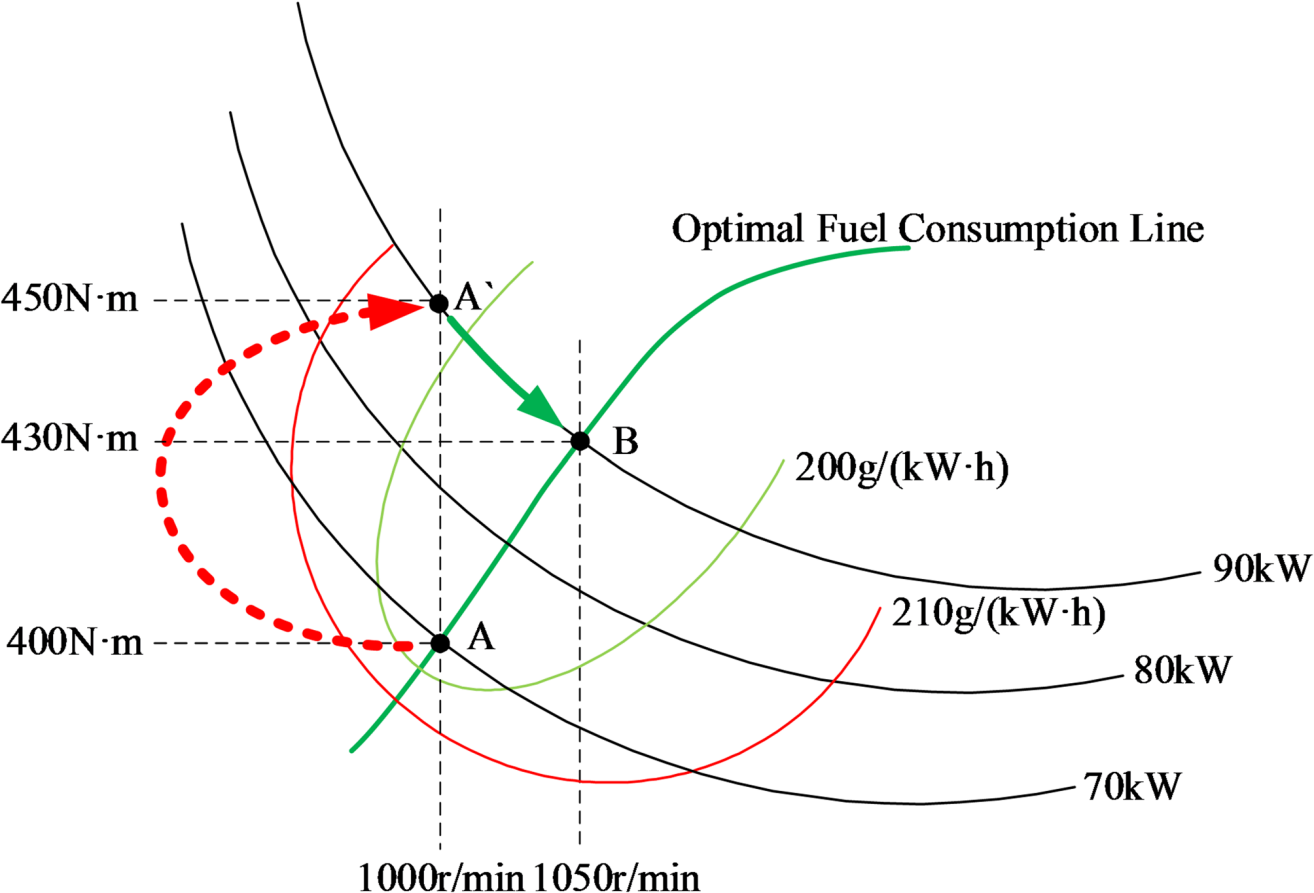
Principle of engine operating point transition.

Based on the proposal of the optimal fuel consumption curve, this paper simplifies the engine control process and shifts the operating point to the optimal point according to the constant power curve under specific operating conditions. As shown in Fig. 8, a schematic diagram illustrates the principle of engine operating point transition. Initially, the engine operates at point A (1000 r/min, 400 N·m), with a speed of 1000 r/min and torque of 400 N·m, located within a fuel consumption rate of 200 g/(kW·h). At a certain moment, when encountering a load, the electronically controlled engine’s torque and speed change accordingly. After stabilization, the speed remains constant^22^. Subsequently, the operating point shifts along the red speed response curve to point A’ (1000 r/min, 450 N·m). However, at this point, the fuel consumption rate increases to 210 g/(kW·h), leading to higher fuel consumption. To improve fuel efficiency while meeting the external load demand, the operating point A’ can be shifted to point B (1050 r/min, 430 N·m) based on the power required for the current load. At point B, the engine operates within the 210 g/(kW·h) fuel consumption rate and continues to function on the optimal fuel consumption line, achieving both load accommodation and fuel efficiency.

After identifying the power, the challenge lies in achieving rapid and accurate operating point switching. With the high-pressure common rail fuel system of this engine, precise fuel injection is achieved, leading to more complete combustion. Additionally, referring to the method in Ref^7^a maximum acceleration-based control strategy is used to ensure that the engine speed quickly reaches the target value during operating point switching. When transitioning from a low operating point to a high operating point, the pump control current is reduced to decrease the pump’s absorbed torque, increase acceleration, and rapidly raise the speed. Conversely, when transitioning from a high operating point to a low operating point, the pump control current is increased to enhance the pump’s absorbed torque, maximize reverse acceleration, and quickly lower the speed.

**Fig. 9:**
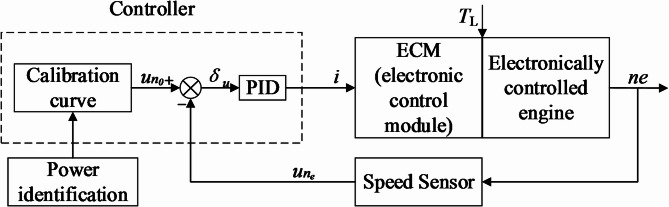
Control strategy for engine operating point.

To ensure that the engine continuously operates along the optimal fuel consumption curve, its operating points must be controlled, as shown in the control strategy in Fig. 9. The four power-speed curves are calibrated and stored in the controller. When the system detects a power value, the optimal speed *n*_0_ corresponding to that power is obtained by looking up the curve. This optimal speed *n*_0_ is set as the target speed. The measured speed *n*_e_, and the deviation *u*_*ne*_ is formed. The controller then converts the deviation into voltage and current signals, considering the torque feedback from the load. The controller adjusts the engine’s speed in real-time through the ECM (Engine Control Module) until the target speed is reached. At this point, the engine operates at the optimal working point, where both torque and speed are optimized for fuel efficiency at the given power level.

### Hydraulic system performance optimization

Regarding the performance optimization of the hydraulic system, the goal is to maximize its efficiency at any given time. Cheng^23^ identified compound parameters based on experimental data and derived a complete efficiency model for hydraulic pumps and motors. By maintaining the hydraulic pump and motor operation within the medium-to-high displacement range under various working conditions, the overall transmission efficiency of the CVT system was improved, thereby enhancing energy efficiency.

**Fig. 10:**
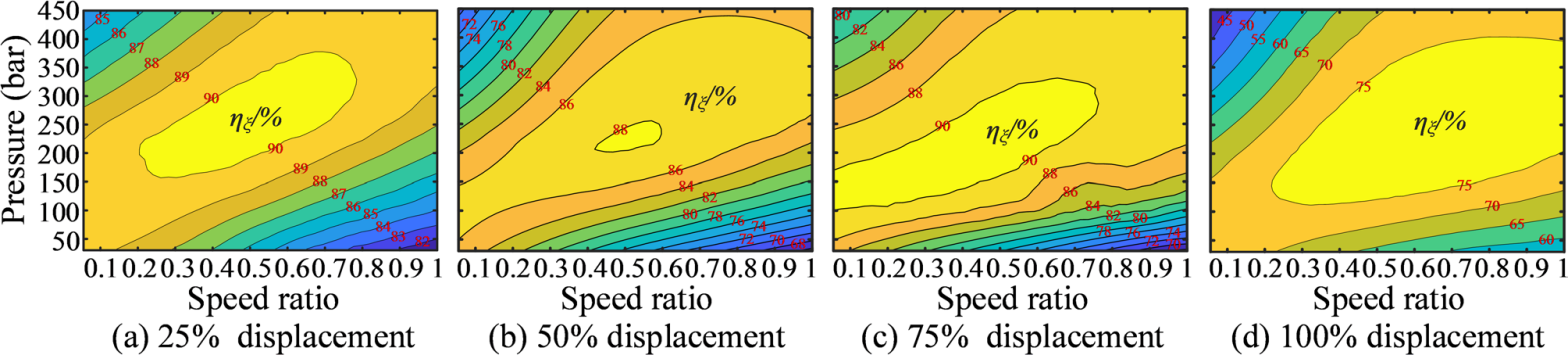
Pressure-speed-efficiency curves at different pump displacement.

**Fig. 11:**
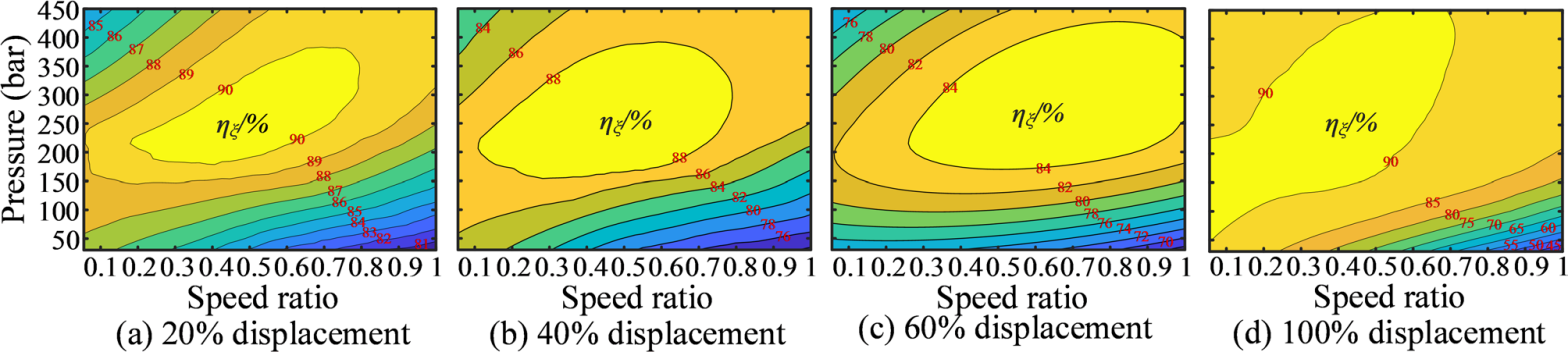
Pressure-speed-efficiency curves at different motor displacement.

Similarly, in this study, based on experimental data, the pressure and speed efficiency curves of the selected pump and motor under different displacements were plotted, as shown in Figs. 10 and 11. The key is how to control the displacements of the pump and motor to achieve constant-speed cruising while coordinating the two to realize optimal efficiency under specific operating conditions. These efficiency curves are critically important in the solution process.

**Fig. 12:**
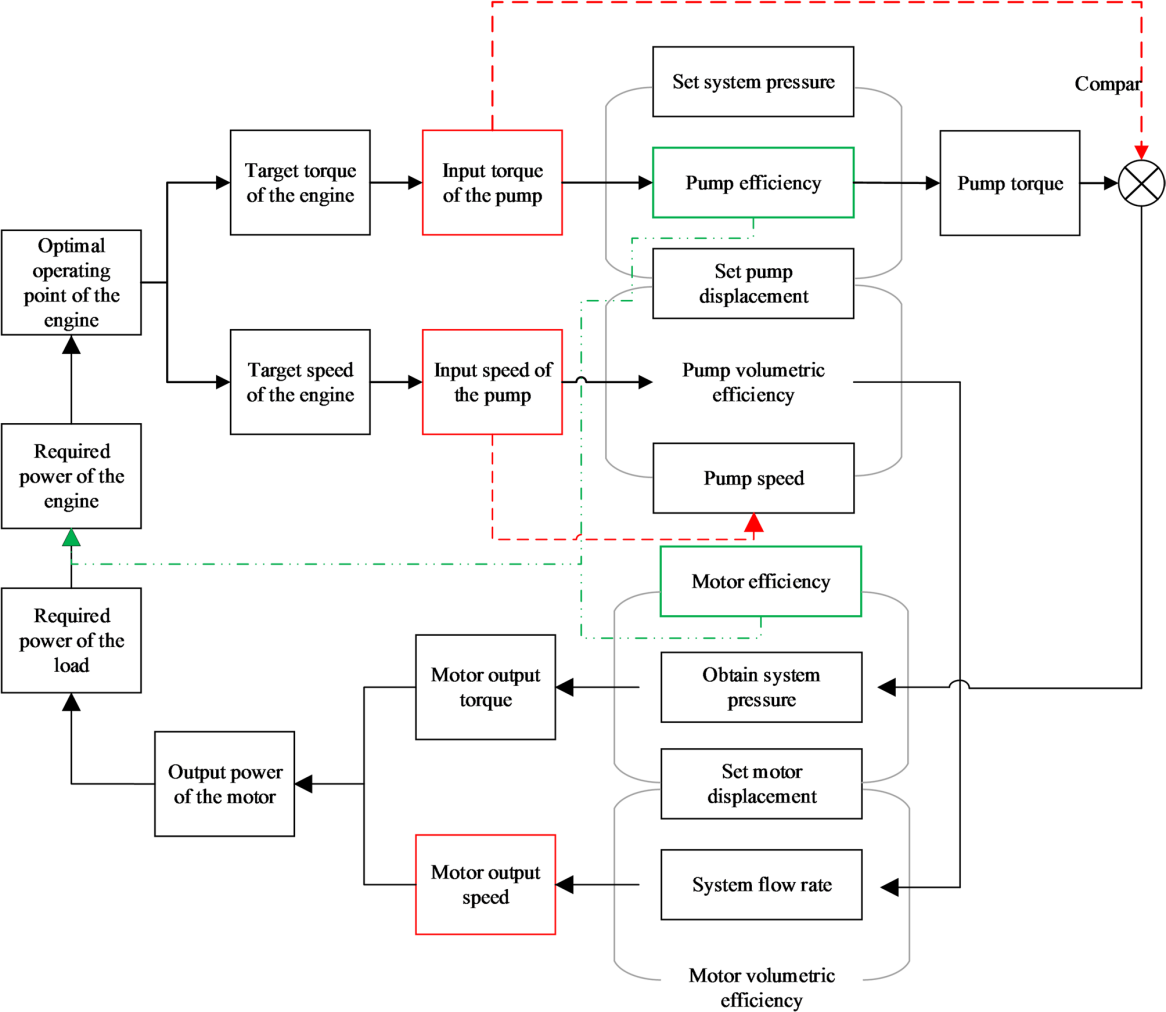
Flowchart for solving the optimal efficiency displacement combination of pumps and motors.

Assuming the engine power is 123 kW and the pump speed is 1600 r/min, to maintain a vehicle speed of 1 m/s, the motor speed needs to be approximately 914.5 r/min, according to the total transmission ratio and track radius used in the bulldozer experiment. Using the designed program, the pump and motor displacement combinations can be calculated for a fixed vehicle speed and the same engine operating point. From these combinations, the optimal efficiency can be identified. The solution flowchart is shown in Fig. 12, and the specific solution process is as follows:

1. Extracting the pump’s output flow rate:

First, set the pump displacement. The pump displacement percentage ranges from 25 to 100%, with the intermediate 75% divided into equal segments. For each segment, the output flow rate is calculated iteratively by multiplying the pump input speed by the volumetric efficiency and the corresponding displacement ratio. Through this process, the system’s flow rate is effectively extracted.

2. Extracting the pump’s efficiency:

The process begins by identifying the two efficiency curves between which the current pump displacement lies. Next, the corresponding efficiency values at the relevant speeds and pressures are located from the charts. The current pump efficiency is then calculated using a length ratio-based interpolation method. Specifically, first set the system pressure, which ranges from 5 MPa to 45 MPa and is divided into intermediate kbar levels. Then, calculate the input torque of the hydraulic pump using the following formula:

8 $${T_0} = \frac{{{p_0}{V_0}}}{{2\pi {\eta _0}}}$$

where *T*_0_ represents the input torque of the hydraulic pump; *p*_0_ represents the currently set pressure; *V*_0_ represents the currently set displacement; *η*_0_ represents the currently calculated mechanical efficiency of the pump.

Subtract this torque value from the pump input torque and use logical indexing to find positive values. If positive values exist, return the index of the first positive value; otherwise, return an empty array. If no positive values are found, return null. Using this method, the pressure value can be extracted. With the pressure value, the pump efficiency can also be extracted. Finally, using a method similar to step 2, the motor efficiency is calculated, and the total efficiency of different pump and motor displacement combinations is obtained.

**Fig. 13:**
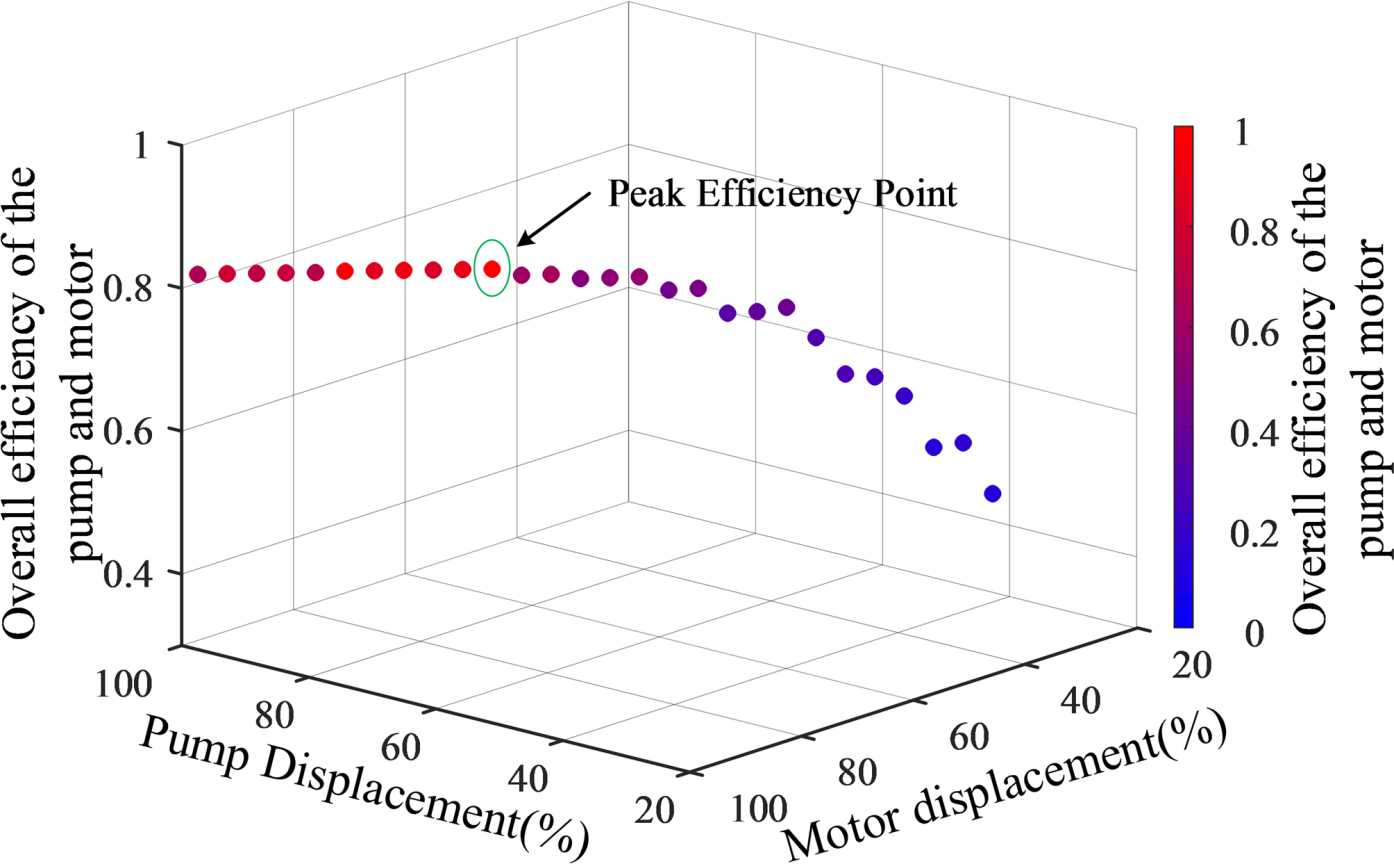
Overall efficiency of different pump and motor displacements.

As shown in Fig. 13, the solution results are presented, with the group achieving the highest overall efficiency selected: hydraulic pump displacement at 75%, hydraulic motor displacement at 72.8%, and an overall efficiency of 0.81. These solution parameters are then input into the simulation system for phase-specific simulation. This method enables the determination of the optimal working point for each load condition, resulting in a set of pump and motor displacement combinations and the corresponding optimal efficiency.

**Fig. 14:**
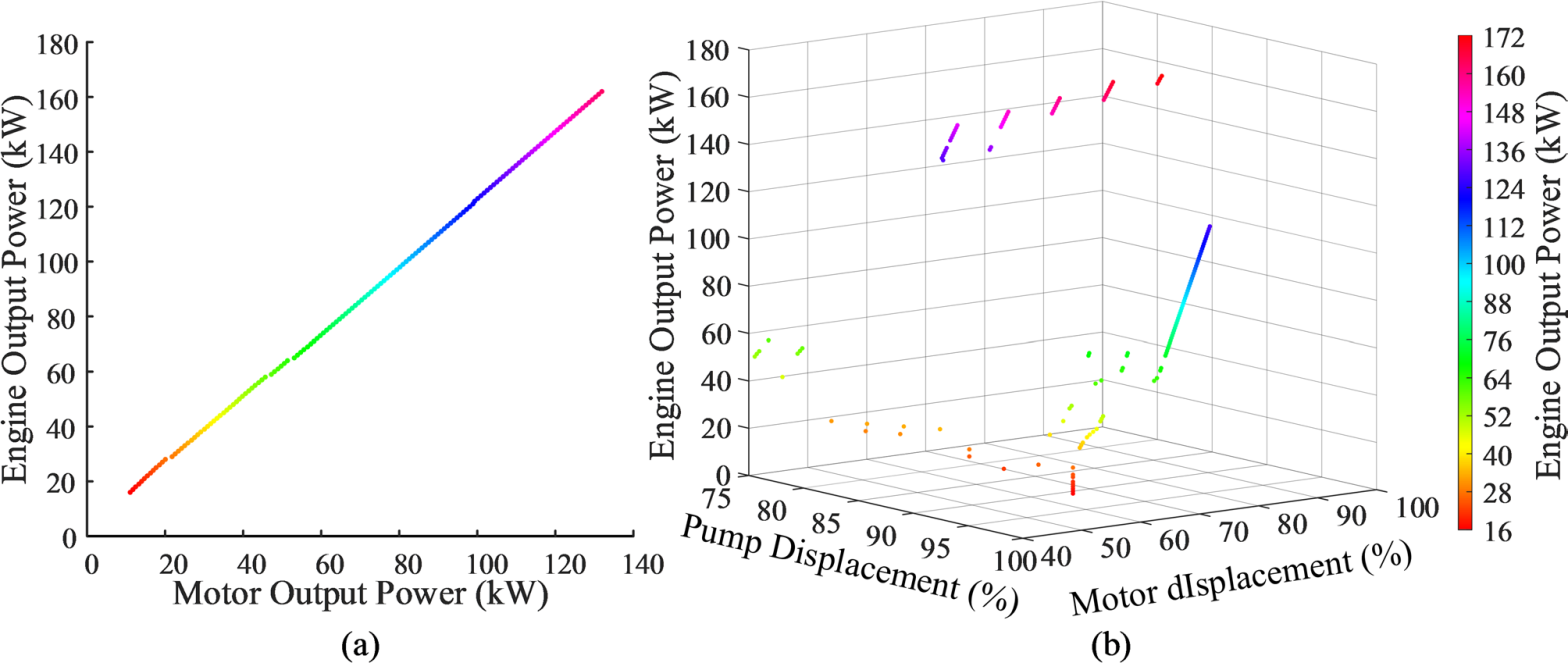
Coordinate system model: (**a**) engine output power versus motor output power; (**b**) engine output power versus the pump-motor displacement combination.

Organize the data and establish a coordinate system model of engine output power versus motor output power, as shown in the Fig. 14a, and fit a function. In the model, this is represented as a 1-D lookup table. This allows for rapid querying of the required engine output power as the motor output speed changes with the load, achieving power identification. Next, establish a coordinate system model of engine output power versus the pump-motor displacement combination, as shown in the Fig. 14b. It is important to note that this model is a scatter plot, so function fitting cannot be performed. Therefore, the determined engine output power must be rounded to ensure accurate querying of the pump-motor displacement combination in the lookup table.

This solution method not only determines the displacement signals for the pump and motor but also minimizes system energy loss while maintaining the motor’s speed. As a result, it ensures that the engine’s transmitted power is fully absorbed by the hydraulic system, thereby achieving power matching between the engine and the load.

## Co-simulation and experiment

### Engine and control strategy modeling

The engine model is crucial in studying power matching in the powertrain system. An accurate engine model can take the actual operating conditions of the engine as inputs for the simulation, transmitting them to the powertrain system’s power matching model. This allows the simulation to closely reflect real operating conditions and ensures that the results of the simulation are more aligned with the actual operation.

The speed of the electronic fuel injection (EFI) diesel engine is controlled in a typical closed-loop manner. The speed control principle involves using the actual speed as feedback and setting a target speed. The PID algorithm is employed to simulate the engine’s electronic control unit (ECU), which adjusts the torque that can be provided. The difference between the target and actual speed is used as an error to guide the ECU in controlling the fuel injection, achieving the goal of speed control.

During the engine speed adjustment phase, to cope with varying loads, the EFI diesel engine automatically adjusts the fuel injection. Based on the torque balance equation, a dynamic model for speed and torque can be established.

9 $${T_{provide}} - {T_{load}} - {T_{emp}} - D\frac{\pi }{{30}}{n_{\text{t}}} = J\frac{\pi }{{30}}\frac{{dn(t)}}{{dt}}$$

where *T*_*provide*_ represents the torque provided by the engine; *T*_*load*_ represents the load torque; *T*_*emp*_ represents the no-load torque; *D* represents the friction coefficient; *n*_*t*_ represents the actual engine speed; *J* represents the engine’s moment of inertia.

Definition of input:

10 $${x_i}(t) = {T_{provide}} - {T_{load}} - {T_{emp}}$$

Definition of output:

11 $${x_o}(t) = {n_{\text{t}}}$$

By combining Eqs. (11) and (12) and applying the Laplace transform, the transfer function of the controlled system can be obtained as:

12 $$G(s) = \frac{{{X_o}(s)}}{{{X_i}(s)}} = \frac{{30}}{\pi }\frac{1}{{Js + D}}$$

This study is primarily energy-saving oriented, so it is necessary to establish a fuel consumption model for the engine to verify the feasibility of the control strategy. The engine’s fuel consumption characteristic curve is fitted using interpolation surface fitting. The resulting fuel consumption model is then incorporated into the engine model as a 2-D table module for easy fuel consumption calculation.

**Fig. 15:**
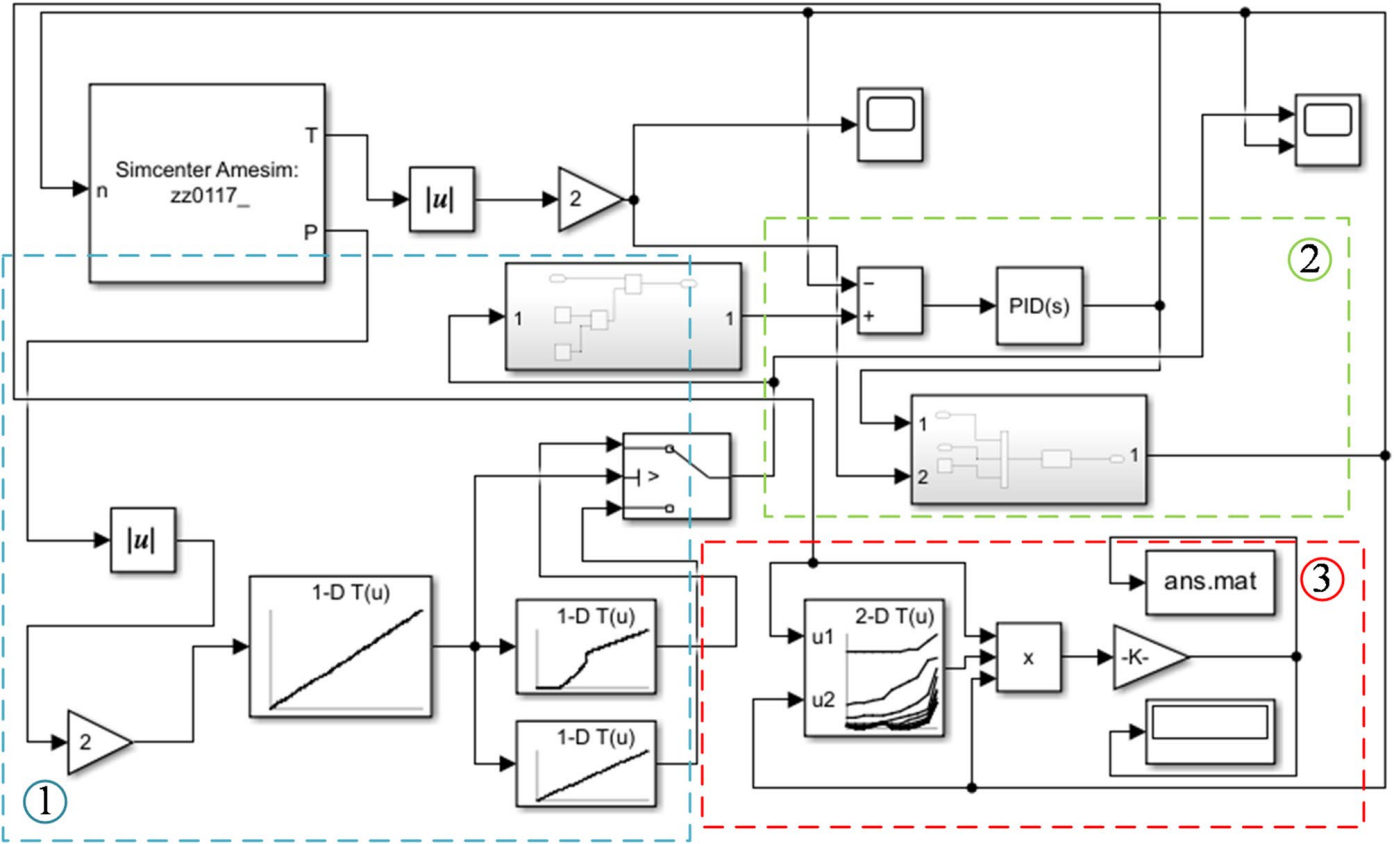
Engine and control model.

Based on the above content, the engine model is established as shown in Fig. 15. According to the functions of each module, the system can be divided into the following modules: (1) Operating point identification module; (2) Operating point transfer control module; (3) Fuel consumption calculation module.

### Hydraulic drive system model

**Fig. 16:**
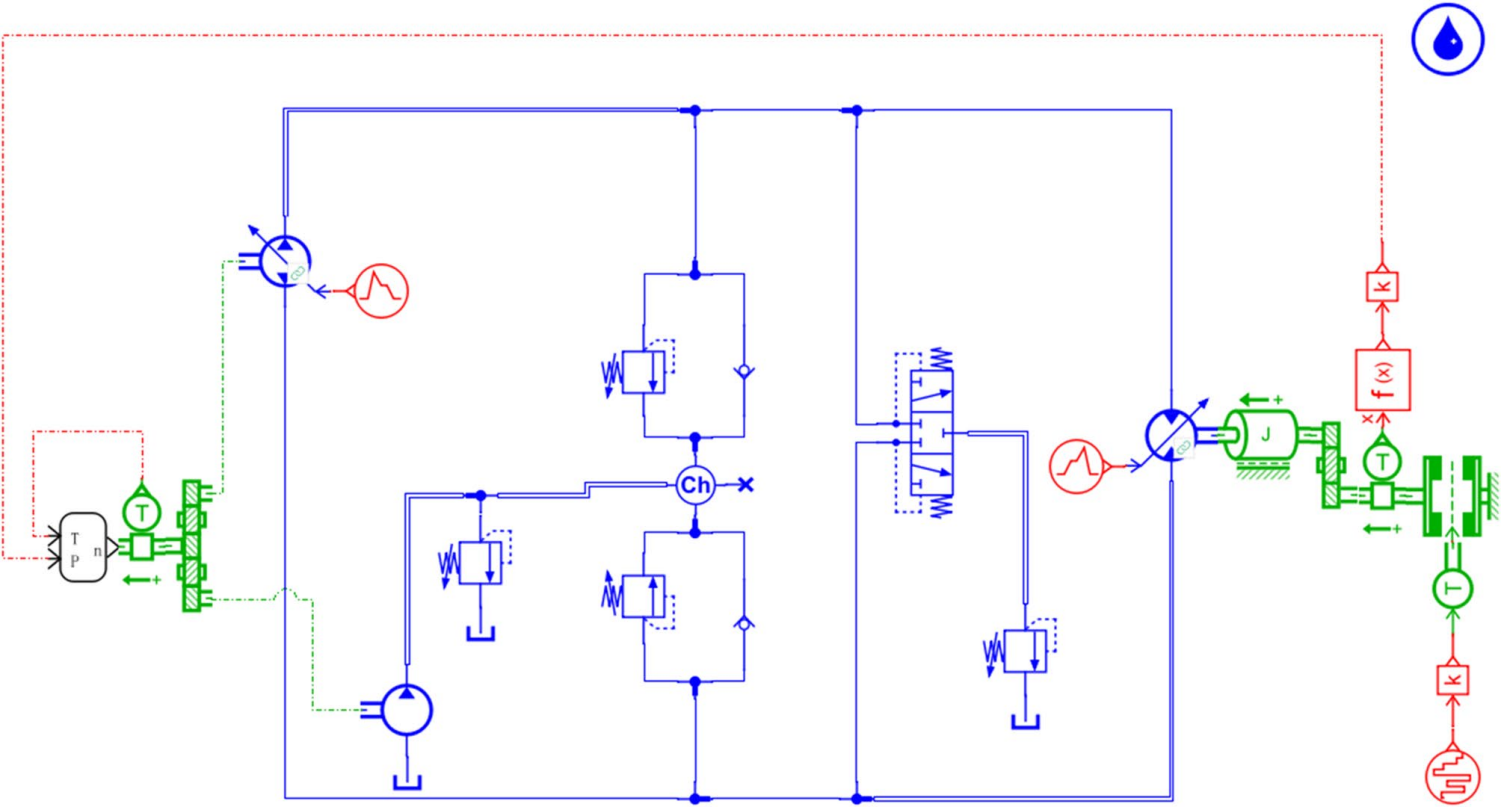
Hydraulic drive system model.

To facilitate the simulation, based on the schematic diagram in “Analysis of hydraulic transmission system operating characteristics”, only a single-sided hydraulic drive system is considered. Half of the equivalent load is taken as the single-side load, and a simulation model of the single-sided hydraulic drive system is established, as shown in Fig. 16.

The variable pump and variable motor models both select sub-models that account for volume and mechanical losses. In the efficiency settings, different efficiency values for pressure and speed at various displacements are input based on experimental data. As previously analyzed, both the hydraulic pump and hydraulic motor are current-proportional variable displacement components, so the control systems of the pump and motor are simplified by replacing them with control signals.

In the pressure-limiting model, the safety valve in the high-pressure circuit is set to a pressure of 450 bar. This setting ensures that the hydraulic system operates at the maximum allowable pressure, preventing damage to components when encountering heavy loads, such as tree roots or large rocks, during operations. If the pressure is cut off, the system’s pressure drops instantaneously, causing the engine to idle. Once the pressure sensor detects the system pressure, the controller adjusts the operating point to a low-power, low-speed state after power conversion, preventing the engine speed from rising continuously and causing a stal.

In the simulated load model, the motor output shaft is connected to a rotating load dynamics model. The subsequent reducer model can be set with transmission efficiency, with internal values configured based on experimental data, in an attempt to simulate the additional energy losses that should be considered in the actual working conditions of the powertrain system. The final component is the rotating friction torque generator, which can model random load variations using a random step signal generator to set the torque experienced by the system.

The output of the engine model is connected to a rotational node sub-model, which simulates the connection of one input shaft to two or more output shafts. In this sub-model, the torque at the interface connected to the engine is the sum of the torques at the other two interfaces, while the rotational speed of all three interfaces is the same. Since the power required by the oil pump itself is relatively low, in this theoretical simulation, the torque is assumed to be evenly distributed to the variable hydraulic pump. Two pressure sensors are placed at both ends of the pump, allowing the pump pressure to be calculated based on the pressure differential. The feedback signals from the pressure and flow sensors are then input into the engine model, facilitating the calculation of the required engine power and torque.

### Co-simulation

**Fig. 17:**
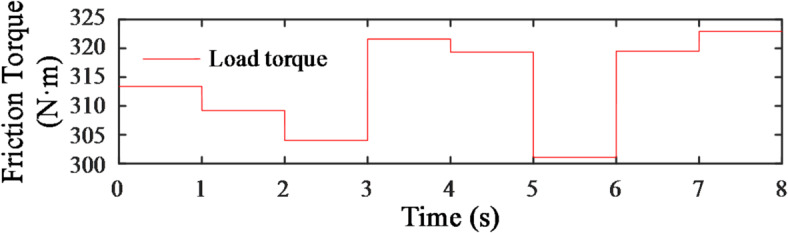
Analog load change curve.

During bulldozing operations, the primary external resistances acting on the machine are the dozing resistance and the sliding resistance required to drive the machine body. The former involves sliding friction, with a coefficient of 0.14 under soft soil conditions. Based on the transmission ratio and track radius, the sliding resistance torque exerted on the hydraulic motor is approximately 226 N·m. The latter, as shown in the Fig. 17, is set as a step torque signal with a mean value of about 310 N·m. These two components together constitute the total bulldozing load, which is applied to the simulated load module.Each segment has an interval of 1 s, and the sampling interval of the power sensor is also set to 1 s. The total simulation time is 8 s.

**Fig. 18:**
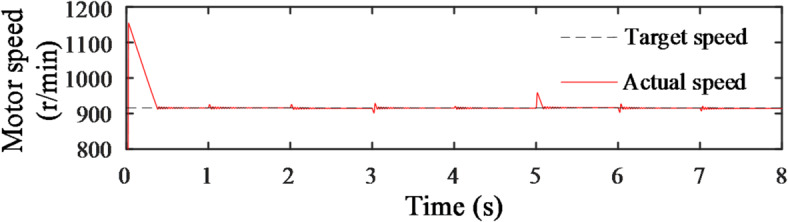
Motor speed change curve.

As shown in Fig. 18, the curve represents the variation in the hydraulic motor speed. In each load change phase, there is a brief fluctuation, which occurs because the engine adjusts its operating point based on the load variation. The changes in speed affect the flow within the system, but it quickly stabilizes to the target speed of 914.5 r/min due to the displacement adjustment of the pump and motor. The bulldozer’s speed is calculated based on the motor output shaft speed, with the final result being a vehicle speed maintained around 1 m/s.

**Fig. 19:**
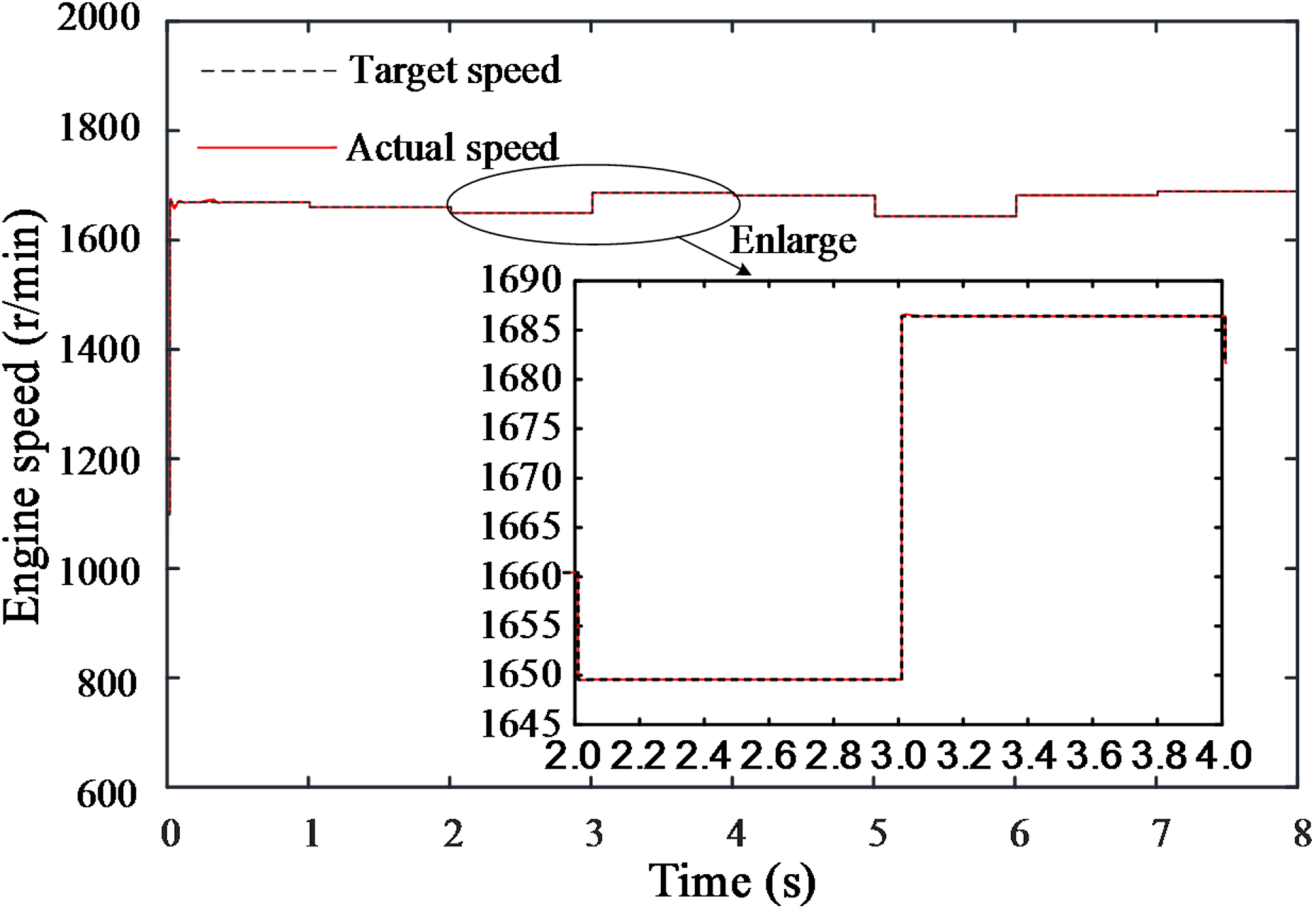
Engine speed change curve.

**Fig. 20:**
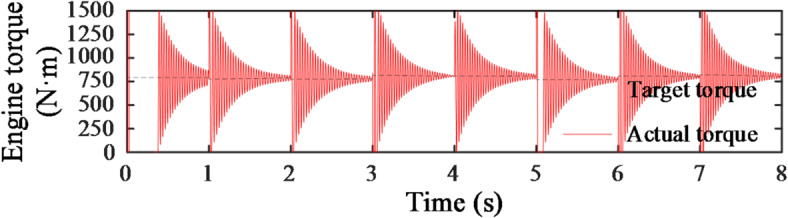
Engine torque change curve.

As shown in Figs. 19 and 20, the curves represent the changes in engine speed and torque during each load variation phase. It is evident that, in each phase, the engine speed adjusts quickly, reaching the target value within 0.1 s with minimal fluctuation. Although the engine torque fluctuates more significantly, it stabilizes and remains close to the target value after some corrective adjustments.

**Fig. 21:**
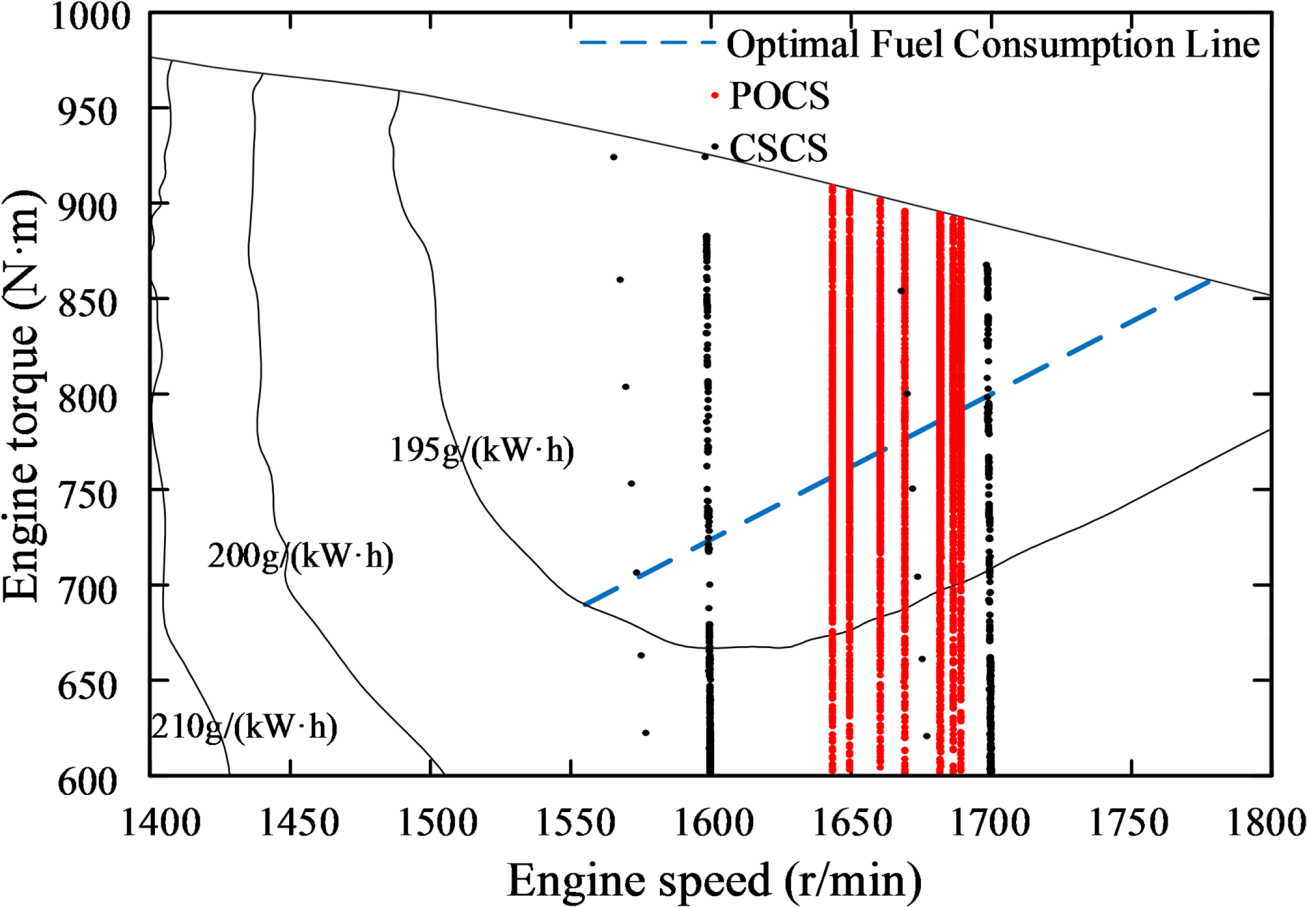
Comparison of engine operating point changes under different control strategies.

To further observe the distribution of the engine’s operating points, all the operating points are plotted on the universal characteristic curve, as shown in Fig. 21. Additionally, the operating points for two different constant speed control strategies(CSCS) are included. The method involves modifying the engine model’s input to a constant target speed. The pump and motor displacement are solved in the same way as in this study. Finally, the distribution of the operating points is observed while maintaining the motor speed.

It is evident that the operating points of both control strategies shift along the speed regulation curve. The standard deviations of the working points, calculated relative to the optimal fuel consumption line, are reduced by 8.3%(1600 rpm) and 13.6%(1700 rpm) under POCS compared to CSCS, clearly demonstrating that the proposed control strategy effectively concentrates the engine working points. This verifies the effectiveness of the former control strategy. Comparing fuel consumption, as shown in Table 3, the relative fuel savings of the former strategy compared to the latter are 6.4% and 3.7%, respectively.

**Table 3:** Fuel consumption under different control strategies.

Strategies	Fuel consumption	
	Value/mL	Relative value/%
CSCS (1700 rpm)	47.59	100
CSCS (1600 rpm)	46.28	97.2
POCS	44.50	93.5

**Fig. 22:**
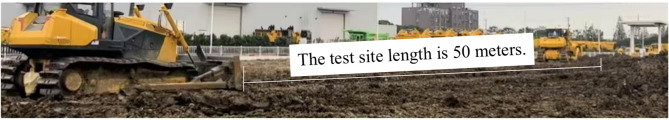
Pre-test preparation.

**Fig. 23:**
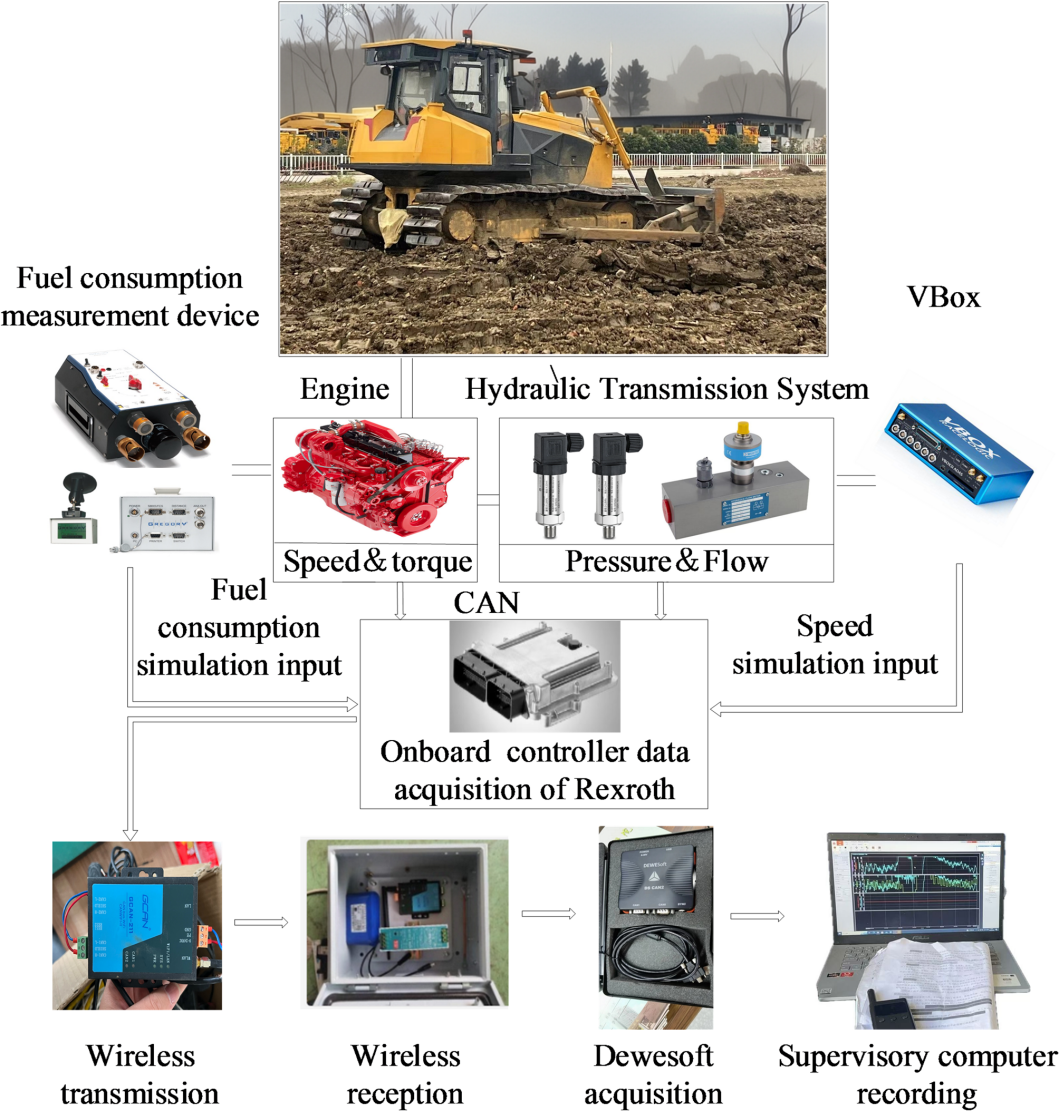
The test system for the bulldozer.

The test method follows the guidelines of GB/T 6375 − 2008 Earth-moving machinery−Method of test for the measurement of drawbar pull and GB/T 35,202 − 2017 Earth-moving machinery−Crawler tractor-dozer−Test methods along with other relevant standards^18^. As shown in Fig. 22, the test section is approximately 50 m long and 6 m wide. The road surface consists of cohesive soil, providing moderate adhesion and low rolling resistance. The longitudinal slope of the road does not exceed 0.4%, the transverse slope is no greater than 2.5%, and the wind speed is limited to no more than 6 m/s. Furthermore, based on the previously discussed principles of the bulldozer hydraulic system and electronic control system, the test system was designed as illustrated in Fig. 23.

The test system utilizes the J1939 protocol to extract engine speed and torque, and GREGORY fuel consumption meter from Germany’s Flow-Tronic company to collect engine fuel consumption data. An encoder is installed on the hydraulic torque converter turbine to extract its speed, while the VBox is used to measure the bulldozer’s vehicle speed. The Dewesoft data acquisition system is employed for data display and storage. Set the bulldozer to conduct a loaded test at a speed of 1 m/s in a 50-meter-long test field by gradually lowering the blade. Compare the CSCS with the POCS.

**Fig. 24:**
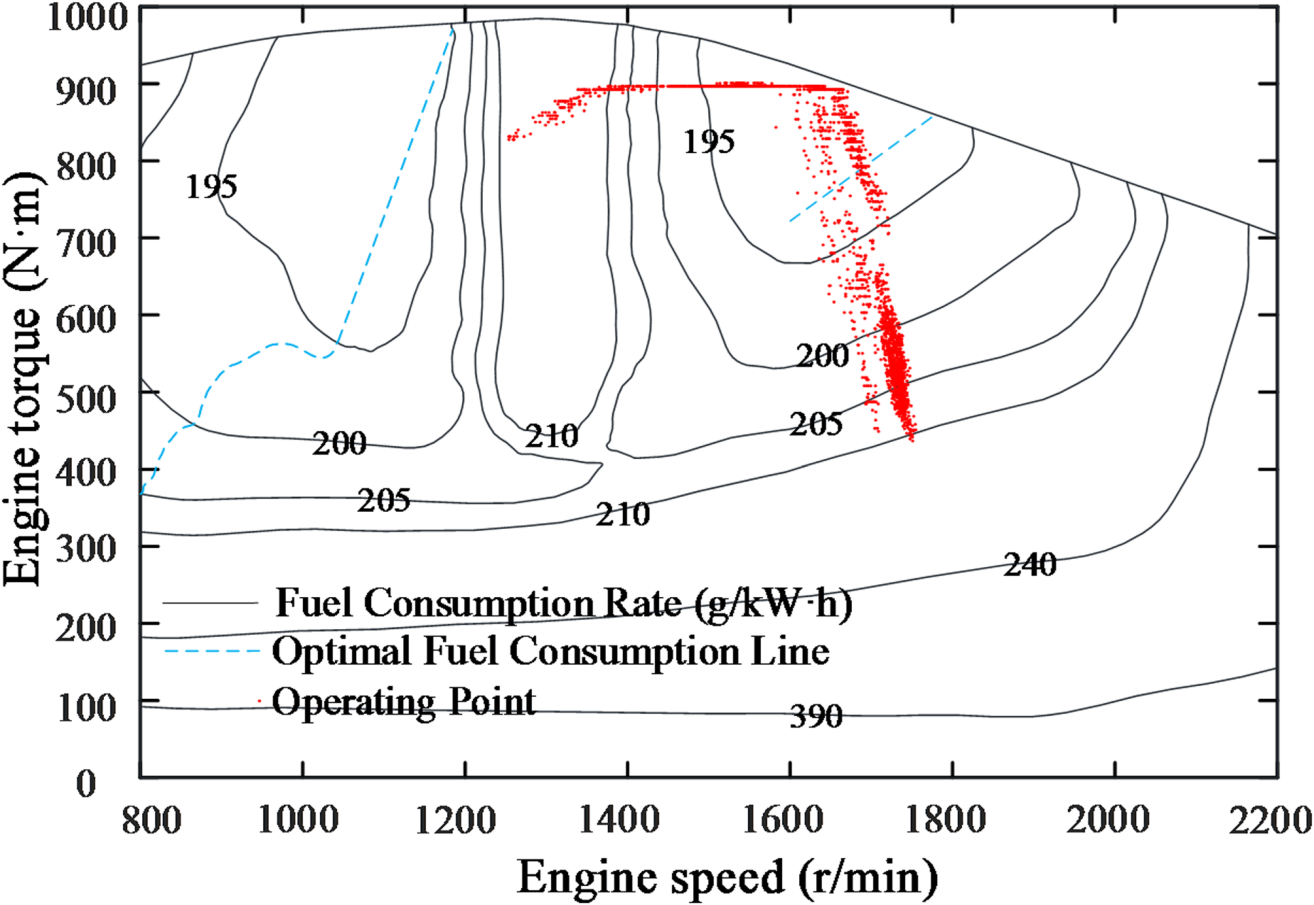
CSCS test operating point distribution.

**Fig. 25:**
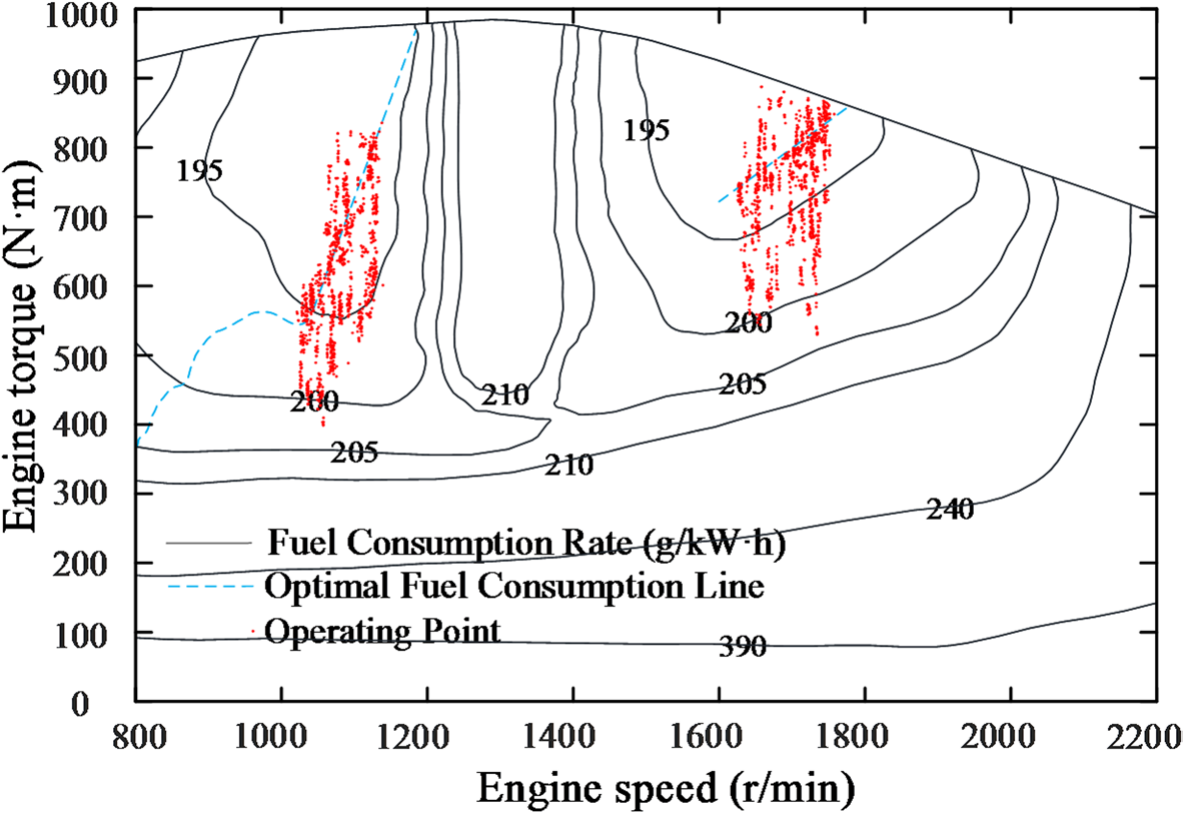
POCS test operating point distribution.

The test working point distributions of the CSCS and the POCS are shown in Figs. 24 and 25, respectively. It is evident that the working points of the former are relatively scattered compared to the latter, with most points not located in the optimal fuel consumption region. Additionally, some points are situated in the transition region between light and heavy loads. In contrast, the latter strategy enables working points to shift according to load recognition, with most points positioned near the optimal fuel consumption line. As shown in Table 4, the latter strategy achieves a relative fuel consumption saving of 4.1% compared to the former, demonstrating significant energy-saving effects.

**Table 4:** Fuel consumption under different control strategies.

Strategies	Fuel consumption	
	Value/mL	Relative value/%
CSCS (1700 rpm)	277.44	100
POCS	266.04	95.9

## Conclusion

From the perspective of bulldozer energy efficiency and operational precision, a performance optimization strategy considering both the engine and the hydraulic system was developed under the framework of constant-speed cruise control. An optimal fuel consumption curve was designed based on the universal characteristics of the engine, enabling dynamic working point adjustment. During the working point transition design, power matching between the engine and load was incorporated. Additionally, a method for determining the optimal combination of pump and motor displacements was proposed to maximize system efficiency, resulting in a POCS based cruise control.Based on a coordinated throttle and displacement control approach, the proposed method identifies the optimal displacement combination to maximize hydraulic system efficiency. A constant-speed cruise control strategy that considers engine-load power matching and optimal energy transfer was established. The control strategy was modeled and experimentally validated, comparing working point distributions and fuel consumption results between traditional constant-speed control and the performance-optimized control strategy.

Both simulation and experimental results demonstrated that, compared to the CSCS, the POCS achieved a more concentrated distribution of engine working points. The working points transitioned dynamically along the optimal fuel consumption curve according to load variations, leading to significant improvements in fuel economy. Such improvements in fuel efficiency can lead to substantial cost savings over the lifetime of the equipment and contribute to promoting more sustainable and economical practices in the construction machinery industry.

This study validated the feasibility of the proposed POCS based cruise control under specific and relatively stable working conditions. However, the model and control strategy have yet to be tested under highly dynamic or extreme load variations. Future work should consider more complex and rapidly changing load conditions, such as those encountered in variable terrains or automated operations. Moreover, more advanced control algorithms should be applied to achieve greater precision and intelligence in system response, further enhancing the overall energy efficiency and operational performance of hydrostatic bulldozers. In addition, future studies could incorporate emissions (CO, NOx, HC, PM) control into the performance optimization strategy to enable a more comprehensive assessment of environmental impacts^24^.

## Data Availability

The data that support the findings of this study are available from [LIUGONG CHANGZHOU MACHINERY CO.，LTD.] but restrictions apply to the availability of these data, which were used under license for the current study, and so are not publicly available. Data are however available from the corresponding author upon reasonable request and with permission of [LIUGONG CHANGZHOU MACHINERY CO.，LTD.].

## References

[1] Muniasamy, A. Machine learning for smart farming: a focus on desert agriculture. In *2020 International Conference on Computing and Information Technology*. **1441**(01), 438–442 (2020).

[2] Guzman, N. H. C., Mezovari, A. G., Yan, Y. & Petersen, M. L. An IoT-based prototype of a driverless bulldozer. In *2019 15th International Conference on Distributed Computing in Sensor Systems (DCOSS)*. IEEE. 291–296. (2019).

[3] Shi, Y. *Study on Working Stage Identification and Staged energy-saving Control of Hydraulic Excavator* (Central South University, 2022).

[4] Yuan, Y. et al. Research on Electric Bulldozer Straight Driving Stability. In *2019 3rd Conference on Vehicle Control and Intelligence (CVCI)*. IEEE. 1–6. (2019).

[5] Zhao, C. et al. Design and experiment of cruise control system for hydrostatic transmission tractor. *Trans. Chin. Soc. Agric. Mach.* **52** (4), 359–365 (2021).

[6] He, Q., Chang, Y. & Hao, P. The study of the correspond control between constant power and variable power of hydraulic excavator. *Constr. Mach.* **2006**(03):0055–0004 .

[7] Gao, Y., Feng, P., Peng, B. & Qiu, Q. Stage-based power matching control of hydraulic excavator. *J. Harbin Eng. Univ.* **38**(9), 1461–1469 (2017).

[8] Choi, J., Kim, H., Yu, S. & Yi, K. Development of integrated controller for a compound hybrid excavator. *J. Mech. Sci. Technol.* **25**, 1557–1563 (2011).

[9] Kim, H., Choi, J. & Yi, K. Development of supervisory control strategy for optimized fuel consumption of the compound hybrid excavator. *Proc. Institution Mech. Eng. Part. D: J. Automobile Eng.* **226** (12), 1652–1666 (2012).

[10] Wang, X., Zhang, J. & Zhang, R. A Design Approach to Optimal Cruising Control with String Stability Constraint for Vehicle Platoon. In *2021 60th Annual Conference of the Society of Instrument and Control Engineers of Japan (SICE)*. IEEE. Sep; 53–57. (2021).

[11] Shu, F., Yong, H. & Hui, F. Recent development in automatic guidance and autonomous vehicle for agriculture. *Rev. J. Zhejiang Univ. (Agric Life Sci)*. **44** (4), 381–391 (2018).

[12] Wang, Z., Liu, Z., Bai, X. & Gao, L. Longitudinal acceleration tracking control of tractor cruise system. *Trans. Chin. Soc. Agric. Mach.* **49**(1), 21–28 (2018).

[13] He, J. et al. Design and experiment of automatic operation system for rice transplanter. *Trans. Chin. Soc. Agricultural Mach.* **50** (3), 17–24 (2019).

[14] Coen, T., Saeys, W., Missotten, B. & De, B. J. Cruise control on a combine harvester using model-based predictive control. *Biosyst. Eng.* **99** (1), 47–55 (2008).

[15] Susanto, S. & Sunarno, S. Pengendalian Kelajuan Kendaraan Menggunakan fuzzy logic controller (Flc) Pada sistem cruise kontrol. *Indonesian J. Math. Nat. Sci.* **39** (1), 40–44 (2016).

[16] Li, M. *Research on Power Matching of Hydraulic Excavator Under Different Working Conditions* (Jilin University, 2020).

[17] Zhang, Z. et al. Control and simulation of speed sensing for an EFI engine. In *2017 IEEE 3rd Information Technology and Mechatronics Engineering Conference (ITOEC)*. IEEE. October;435–439. (2017).

[18] Qiang, H. et al. Research into Energy-Saving control strategies of a bulldozer driven by a torque converter based on the minimum fuel consumption rate of the whole machine. *Sustainability* **16** (22), 10111 (2024).

[19] Lei, J., Yang, Y., Yu, F. & Shi, S. Design of travel hydraulic system for model GTQ170 fully hydraulic bull dozer. *Constr. Mach. Equip.* **49** (04), 43–47 (2018).

[20] Wei, W., Yu, T. & Yan, Q. Design and application of hydrodynamic efficiency control system for high-power bulldozer with hydro-mechanical transmission. In *Proceedings of the 2010 International Conference on Modelling, Identification and Control.* IEEE. July;865–869. (2010).

[21] Li, M. et al. Design and test of Electric-hydraulic confluence valve in double pump confluence system. *Trans. Chin. Soc. Agricultural Mach.* **49** (09), 353–360 (2018).

[22] Pang, X., Cao, X., Liu, W., Wang, D. & Wang, D. Research on energy saving control strategy of hydraulic excavator based on EFI engine. *Mach. Tool. Hydraulics*. **51** (04), 7–14 (2023).

[23] Cheng, X., Peng, Z. & Jing, C. Efficiency model of hydraulic Pump-Motor for tractor Hydro-Mechanical CVT under all operating conditions. *Trans. Beijing Inst. Technol.* **45** (02), 154–164 (2025).

[24] Milićević, S. & Blagojević, I. Managing fuel consumption and emissions for hybrid electric vehicles through optimization of engine operation. *Therm. Sci.* **00**, 25–25 (2025).

